# Factors Influencing Stream Segregation Based on Interaural Phase Difference Cues

**DOI:** 10.1177/23312165241293787

**Published:** 2024-12-10

**Authors:** Nicholas R. Haywood, David McAlpine, Deborah Vickers, Brian Roberts

**Affiliations:** 1 151895 Department of Clinical Neurosciences, The University of Cambridge, Cambridge, UK; 2 336640 Department of Linguistics, Macquarie University, Sydney, Australia; 3 School of Psychology, 1722 Aston University, Birmingham, UK

**Keywords:** auditory scene analysis, stream segregation, binaural hearing, interaural phase differences, binaural integration

## Abstract

Interaural time differences are often considered a weak cue for stream segregation. We investigated this claim with headphone-presented pure tones differing in a related form of interaural configuration—interaural phase differences (ΔIPD)—or/and in frequency (ΔF). In experiment 1, sequences comprised 5 × ABA– repetitions (A and B = 80-ms tones, “–” = 160-ms silence), and listeners reported whether integration or segregation was heard. Envelope shape was varied but remained constant across all tones within a trial. Envelopes were either quasi-trapezoidal or had a fast attack and slow release (FA-SR) or vice versa (SA-FR). The FA-SR envelope caused more segregation than SA-FR in a task where only ΔIPD cues were present, but not in a corresponding ΔF-only task. In experiment 2, interstimulus interval (ISI) was varied (0–60 ms) between FA-SR tones. ΔF-based segregation decreased with increasing ISI, whereas ΔIPD-based segregation increased. This suggests that binaural temporal integration may limit segregation at short ISIs. In another task, ΔF and ΔIPD cues were presented alone or in combination. Here, ΔIPD-based segregation was greatly reduced, suggesting ΔIPD-based segregation is highly sensitive to experimental context. Experiments 1–2 demonstrate that ΔIPD can promote segregation in optimized stimuli/tasks. Experiment 3 employed a task requiring integration for good performance. Listeners detected a delay on the final four B tones of an 8 × ABA– sequence. Although performance worsened with increasing ΔF, increasing ΔIPD had only a marginal impact. This suggests that, even in stimuli optimized for ΔIPD-based segregation, listeners remained mostly able to disregard ΔIPD when segregation was detrimental to performance.

## Introduction

Sound sources often generate acoustic energy overlapping in frequency and time. To construct a meaningful representation of individual sources, including their identity and location, the auditory system must assign those elements corresponding to a given source from the mixture of acoustic signals arriving at the ears and separate them from elements corresponding to other sources. This process, often referred to as “auditory scene analysis” (Bregman, 1990), can arise through grouping by shared features—e.g., common onset time, spectral proximity, or harmonic relations—and separating them from other sources or indistinct background features to form distinct auditory “streams”. Sequential auditory streaming can be explored by presenting repeating sequences of alternating “A” and “B” sounds that differ on one or more acoustic dimensions. “A” and “B” subsets may be grouped together as a single perceptual object or stream (“integration”) or, alternatively, the subsets may be heard as two separate streams (“segregation”). When hearing integration, listeners perceive a single stream that changes in perceptual characteristics—for example, when sounds differ in frequency the stream is perceived as moving back and forth in pitch.

Stream segregation is most often studied using sequences of diotic (identical at the two ears) pure tones with a frequency difference (ΔF) between the A and B subsets. In such cases, factors influencing segregation, two of which are relevant here, are well described. First, segregation becomes more likely as ΔF is increased (Bregman & Campbell, 1971; Miller & Heise, 1950; van Noorden, 1975). More generally, spectral differences between sounds are a robust cue for stream segregation (e.g., Beauvois & Meddis, 1996; Hartmann & Johnson, 1991; van Noorden, 1975), although segregation can also occur when subsets differ perceptually despite evoking similar patterns of excitation in the cochlea (Cusack & Roberts, 2000; Roberts et al., 2002; Vliegen et al., 1999; Vliegen & Oxenham, 1999). Second, segregation is influenced by the rate at which individual elements of a sequence of sounds are presented such that the tendency to segregate a sequence into separate streams typically decreases when the presentation rate between successive sounds slows (van Noorden, 1975; though see Simon & Winkler, 2018, for an exception to this principle when brief sounds are presented extremely rapidly in sequence). One way to manipulate the presentation rate is by varying the interstimulus interval (ISI; the silent period between successive sounds). When sound duration is held constant, increasing the ISI results in a slower presentation rate—i.e., an increase in onset-to-onset time between successive sounds.

Another potential factor supporting stream segregation is source location. Differences in the “ear of entry” between A and B sounds are known to be a strong cue for segregation (i.e., monaural left vs. monaural right: Boehnke & Phillips, 2005; Hartmann & Johnson, 1991). However, the influence on segregation of auditory spatial cues *per se*—differences in the intensity and timing of the sounds at the two ears arising from their location on the horizontal plane—is less well understood than the influence of spectral differences. Indeed, binaural cues are reported as being relatively less potent cues for stream segregation: While interaural level difference (ILD) cues can promote segregation, they are less effective than ear-of-entry differences (Boehnke & Phillips, 2005). Moreover, interaural time difference (ITD) cues appear less effective still and are often considered a weak or limited cue for stream segregation (Moore & Gockel, 2012), despite being a strong cue for source location in human listeners. In contrast, it is known that sudden *changes* in ITD are highly effective at resetting prior build-up in the tendency for stream segregation, in both subjective (Rogers & Bregman, 1998) and objective (Roberts et al., 2008) listening tasks.

Several studies have explored stream segregation arising when A and B subsets differ in their ongoing interaural timing differences (ΔITD). Considering first “subjective” tasks—those in which listeners directly report their perception of the number of streams present—measures of ΔITD-based segregation provide seemingly conflicting accounts. Boehnke and Phillips (2005; broadband noise stimuli) and Füllgrabe and Moore (2012; pure tone stimuli) both found ΔITD sequences were reported as segregated on only ∼20% of trials, even for large values of ΔITD. In marked contrast, Schadwinkel and Gutschalk (2010, 2011), and Carl and Gutschalk (2013), used complex tone stimuli and found that a low proportion of segregated responses was reported only for the smallest ΔITD tested, and that segregation increased monotonically with the magnitude of ΔITD. As yet, these large across-study differences in subjective ΔITD-based segregation have not been accounted for.

A second category of behavioral measures is referred to as “objective” tasks, meaning those in which responses can be classified as correct or incorrect. Here, tasks can be designed such that stream segregation should either aid or impair the detection or discrimination of given stimulus features. ΔITD can aid segregation in tasks where this is favorable to performance—for example, the detection of a melody or rhythm conveyed by target notes that are interleaved with irrelevant distractor notes is easier when the targets and distractors form segregated perceptual streams (Cusack & Roberts, 2000; Dowling, 1973; Turgeon et al., 2002), and a ΔITD between targets and distractors greatly aids performance in such tasks (Hartmann & Johnson, 1991; Middlebrooks & Onsan, 2012; Sach & Bailey, 2004). However, ΔITD cues appear unable to force “obligatory” segregation in tasks where segregation would harm performance—for example, in specific stimulus arrangements, the detection of a delay imposed on one subset of sounds is improved when both subsets are grouped together (Roberts et al., 2002; Vliegen et al., 1999). In such temporal discrimination tasks, performance deteriorates as ΔF is increased. However, a ΔITD does not typically impair performance much beyond otherwise equivalent diotic conditions (Boehnke & Phillips, 2005; Füllgrabe & Moore, 2012, 2014; although see David et al., 2015; Stainsby et al., 2011). As poor performance is an indication of stream segregation, the data suggest that ΔITD promotes only a modest degree of obligatory segregation.

The current experiments differed from the outlined previous research in that they involved manipulating the perceived lateralization of tonal stimuli by varying their interaural phase difference (IPD) rather than their ITD. Specifically, we explored stream segregation when A and B subsets differed in IPD (ΔIPD). Whereas ITDs arise from a global delay to the sound arriving in one ear, IPDs are created by advancing the phase of a carrier signal in one ear relative to the other. For a sound differing only in IPD, the timing of onset, offset, and all envelope features (e.g., attack, decay) is the same for both ears—hence IPDs do not naturally occur in real-world listening. For the most part, we consider our findings from ΔIPD stimuli to be broadly generalizable to ΔITD stimuli, but instances that merit further consideration—such as the use of IPD cues at different carrier frequencies in experiments 2 (task 2c) and 3—are highlighted below in the relevant sections.

Our initial research question concerned whether envelope shape influenced ΔIPD-based stream segregation. It is well established that ITD/IPD cues present near onset and during segments with rapidly rising amplitude are afforded the greatest perceptual weight for lateralization (Hartung & Trahiotis, 2001; Stecker, 2018; Tollin & Henning, 1998, 1999; Wallach et al., 1949), including in stimuli with dynamic IPDs and dichotic envelope characteristics (i.e., dynamic IPDs from binaural beats; Dietz et al., 2013; Haywood et al., 2021; Stecker & Bibee, 2014). All previous studies of ΔITD-based stream segregation employed symmetrical envelopes with trapezoidal profiles—i.e., those with relatively rapid onset and offset ramps and a period of sustained peak amplitude in between (Boehnke & Phillips, 2005; Carl & Gutschalk, 2013; Füllgrabe & Moore, 2012, 2014; Hartmann & Johnson, 1991; Sach & Bailey, 2004; Schadwinkel & Gutschalk, 2010, 2011; Stainsby et al., 2011). Experiment 1 was designed to explore the extent of ΔIPD-based segregation in stimuli with three distinct envelope shapes: Individual sound elements were presented with either trapezoidal or asymmetric envelope shapes; in the latter case, with either a fast attack and slow release (here “FA-SR,” also known as “damped”) or a slow attack and fast release (“SA-FR,” also known as “ramped”). Listeners reported significantly more ΔIPD-based segregation for the FA-SR conditions, despite the FA-SR and trapezoidal envelopes sharing identical onset ramps. This indicated that another factor besides onset profile influenced segregation. IPD/ITD cues around abrupt offsets can affect the perceived lateralization of a sound, but this effect is modest compared to the influence of these cues near onsets (e.g., Akeroyd & Bernstein, 2001; Diedesch & Stecker, 2015; Haywood et al., 2021; Stecker & Bibee, 2014; Stecker & Brown, 2010; Stecker & Hafter, 2009; Tobias & Schubert, 1959; Zurek, 1980). Instead, we propose that the reduced ΔIPD-based stream segregation observed for the two conditions involving abrupt offset ramps (trapezoidal and SA-FR) arose from binaural temporal integration between tones presented in close succession.

This proposal was explored further in experiment 2 by varying the tone presentation rate, the effect of which on ΔITD- or ΔIPD-based segregation has not previously been studied in detail. We found that, for FA-SR tones of fixed duration, ΔIPD-based segregation increased with increasing interstimulus interval (ISI), in contrast to ΔF-based segregation which decreased with increasing ISI (e.g., van Noorden, 1975). We argue that ΔIPD-based segregation may be influenced by binaural temporal integration such that, for short ISIs, binaural cues between streams (A and B subsets of sounds) are integrated and that this acts to reduce perceived segregation. We also found that when streams contained a combination of ΔIPD and ΔF cues, segregation based on ΔIPD was greatly decreased in comparison to the task in which *only* ΔIPD cues were presented. Finally, in experiment 3, we measured performance in a temporal discrimination task (Roberts et al., 2002; Vliegen et al., 1999) using the optimal parameters observed here for subjective ΔIPD-based streaming. We found that ΔIPD resulted in a small but significant increase in delay detection thresholds, suggesting that ΔIPD promotes only modest segregation in this type of task (consistent with the results of Boehnke & Phillips, 2005; Füllgrabe & Moore, 2012, 2014; Stainsby et al., 2011).

## Experiment 1

### Method

#### Listeners

Twelve normal-hearing listeners were recruited from student and researcher populations at Macquarie University (*n* = 7) and the University of Cambridge (*n* = 5). All subjects provided informed consent, received payment, and demonstrated normal audiometric thresholds (≤20 dB HL for tone frequencies 500 Hz–4 kHz; Macquarie: AS208, Cambridge: Affinity 2.0, Interacoustics, Middelfart, Denmark). The study received ethical approval from the Macquarie University Human Research Ethics Committee (reference number 5201700786) and the Cambridge Psychology Research Ethics Committee (application number PRE.2019.093). Of the twelve listeners, eight were male, and the mean age was 23.3 years (range = 19–31 years).

#### Stimuli

Each trial sequence comprised five ABA– triplets. A and B were both pure tones with a duration of 80 ms. There was a 40-ms ISI (silence) between each tone within the triplet. The longer silence (“–”) was 160 ms. Therefore, the combined duration of the triplet (“ABA–”) was always 480 ms, and the total sequence duration was 2.4 s. The amplitude envelope shape was varied across trials, but the A and B tones always shared an identical envelope within a trial. Three envelope shapes were used. The first was quasi-trapezoidal, with 10-ms ramps at onset and offset and a sustained portion held at peak amplitude for 60 ms (FA-S-FR). The FA-SR envelope comprised a 10-ms onset ramp, followed immediately by a 70-ms offset ramp. The SA-FR envelope was the opposite—a 70-ms onset ramp followed by a 10-ms offset ramp. All ramps were created from raised cosine functions, and all stimuli were presented at 70 dB sound pressure level (SPL).

#### Task 1A: ΔF

The A tones were fixed at 400 Hz, and the B tones were set to 4, 6, or 8 semitones (ST) higher (504, 566, and 635 Hz, respectively). All stimuli were diotic. The combination of three envelope shapes and three ΔFs led to nine unique conditions.

#### Task 1B: ΔIPD

Both A and B tones were set to 400 Hz. In a control case, both A and B tones were diotic (i.e., no ΔIPD). For the remaining three levels of ΔIPD, the A tones were presented with a right-ear-leading IPD, and the B tones with a left-ear-leading IPD of equal magnitude. These values corresponded to ±15°, ± 45°, or ±90° (i.e., ΔIPDs of 30°, 90°, or 180°, respectively). IPDs were created by adjusting the starting phase of the tone in each ear; the onset time and amplitude profile of each tone were identical. Hence, the ITD cues available were present only in the fine structure of the stimuli: IPDs of ±15°, ± 45°, and ±90° corresponded to fine-structure ITDs of ±104, ± 313, and ±625 µs, respectively, which are all within the ecological range for adult humans (roughly ±700 µs—e.g., Kuhn, 1977). The combination of three envelope shapes and four ΔIPDs led to 12 unique conditions.

#### Procedure

Listeners first received a written description of stream segregation for both ΔF and ΔIPD stimuli and were then presented with example ΔF sequences, followed by example ΔIPD sequences. Next, the two tasks were presented in a counterbalanced order across the twelve listeners—six completed task 1A before task 1B, and vice versa for the other six. Each task took under 20 min to complete, and listeners completed the entire experiment within a single testing session lasting under one hour. Listeners were given a short break between tasks.

In both tasks, trials were organized into blocks: A block comprised a single trial of each experimental condition, presented in a shuffled order. In task 1A, there were nine trials per block (3 ΔFs × 3 envelopes), and in task 1B there were twelve (4 ΔIPDs × 3 envelopes). In both tasks, two trial blocks were provided as initial training, the data from which were discarded. The main tasks then each comprised 20 trial blocks (task 1A = 180 trials, task 1B = 240 trials). In an individual trial, listeners heard a single presentation of a condition and were asked to report whether they heard either integration or segregation at the end of the sequence (see, e.g., Haywood & Roberts, 2010). Responses were made via the computer keyboard, and on-screen feedback displayed the chosen response. There was a 1.5-s pause between the keyed response and the automatic start of the next trial.

Stimuli were created and presented using Matlab (R2018). At Macquarie University, sounds were presented over Sennheiser 380 Pro headphones (Hannover, Germany) via an Audio Express sound card (MOTU, Cambridge, MA). Output levels were calibrated using a type 2250 sound-level meter (Brüel and Kjaer, Nærum, Denmark), an RA0045 microphone (GRAS, Holte, Denmark) and a type 43AG ear simulator (GRAS). At the University of Cambridge, sounds were presented over Sennheiser HD-600 headphones via an RME Fireface UCX sound card (Haimhausen, Germany). Output levels were calibrated using an MDO3024 oscilloscope (Tektronix, Beaverton, OR, USA) and headphone sensitivity data. At both sites, listeners completed the experiment in a custom-built double-walled sound-attenuating chamber (IAC Acoustics, Naperville, IL, USA). The research data underlying this publication are available online from a repository hosted by the University of Cambridge (see https://doi.org/10.17863/CAM.112233).

### Results

**Figure 1.**
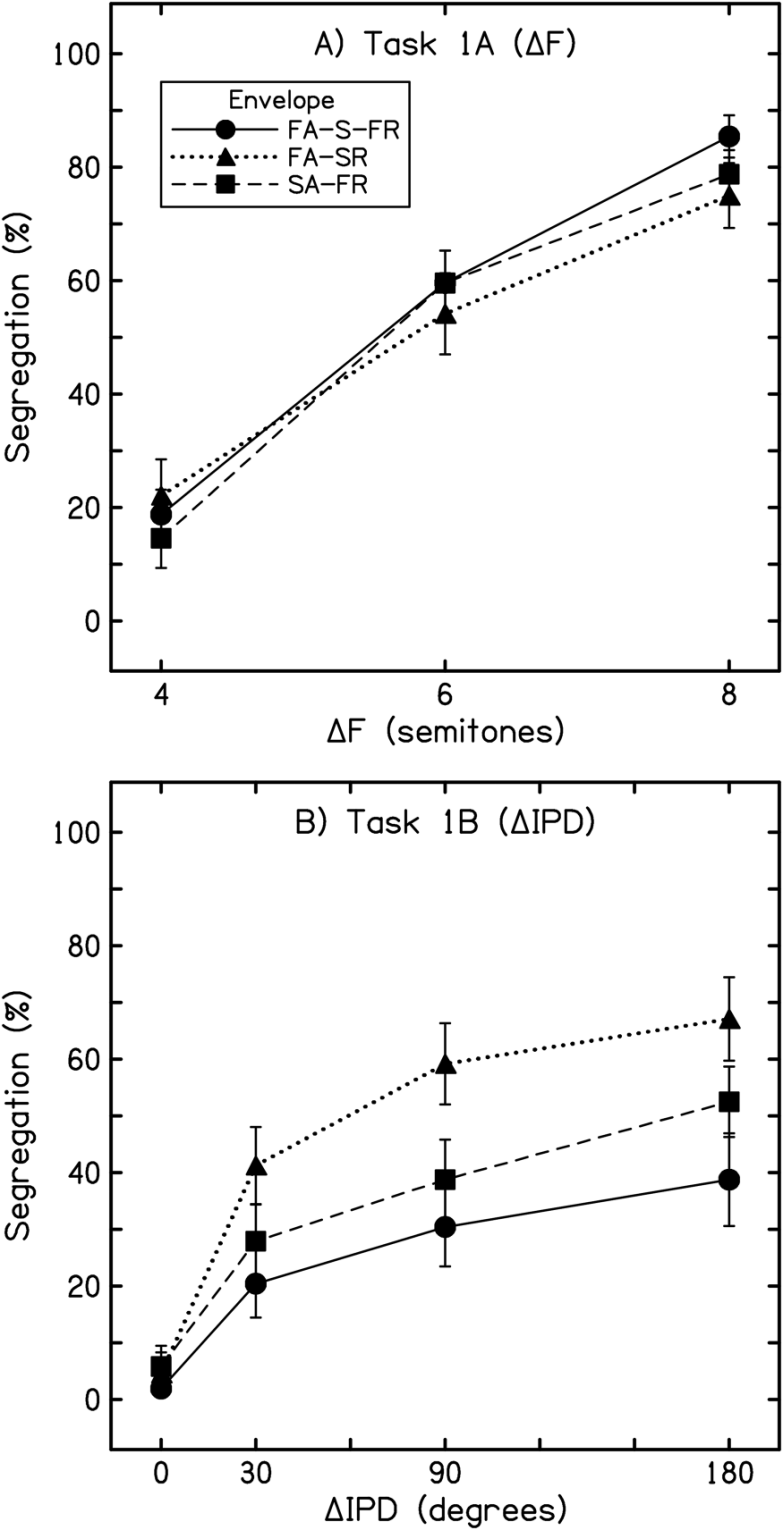
Results from experiment 1. Values are the overall percentage of segregated responses from 12 listeners. Error bars display ±1 intersubject standard error. (A) Results from task 1A: ΔF is plotted along the abscissa, and each trace represents a different amplitude envelope (see inset). (B) Results from task 1B: ΔIPD is plotted along the abscissa. FA-S-FR = fast attack, sustained, fast release (trapezoidal). FA-SR = fast attack, slow release (damped), SA-FR = slow attack, fast release (ramped).

For each listener, responses for each condition were aggregated to yield the percentage of trials heard as segregated, and these percentage values were averaged across listeners to yield the overall percentage of trials heard as segregated (Figure 1). The two tasks—segregation based on ΔF and on ΔPD—were analyzed using a two-way repeated-measures analysis of variance (ANOVA); departures from sphericity were addressed using the Greenhouse-Geisser correction. The measure of effect size reported here is partial eta squared (η*_p_*^2^). All pairwise comparisons (two-tailed) were conducted using the restricted least-significant-difference test (Keppel & Wickens, 2004; Snedecor & Cochran, 1967).

As expected, listeners reported increasing segregation of A and B subsets of tones as the frequency separation (ΔF, task 1A) between tones was increased, and this main effect was highly significant (*F*[1.61, 17.66] = 53.01, *p* < .001, η*_p_*^2 ^= .83). Further, the specific shape of the stimulus envelope had no significant effect on segregation judgments (*F*[1.89, 20.83] = 1.43, *p* = .26, η*_p_*^2 ^= .12), and there was no interaction between frequency separation and envelope shape (*F*[2.17, 23.89] = 1.99, *p* = .17, η*_p_*^2 ^= .15).

For streaming based on ΔIPD (task 1B), segregation increased with ΔIPD, and ΔIPD-based segregation was also strongly influenced by envelope shape. While the symmetrical envelope with a fast attack, sustained component, and fast release (FA-S-FR) was largely ineffectual in promoting segregation, A and B subsets in the FA-SR condition were predominantly reported as being segregated (>50% of all responses) for ΔIPDs of 90° and 180° (59.2% and 67.1% segregation, respectively), despite the absence of any frequency difference between the A and B subsets. The SA-FR envelope was also heard as predominantly segregated with a ΔIPD of 180°, but only marginally (52.5%). Statistically, significant main effects were observed for both ΔIPD (*F*[1.96, 21.51] = 47.58, *p* < .001, η*_p_*^2 ^= .81) and envelope shape (*F*[1.42, 15.58] = 8.51, *p* = .006, η*_p_*^2 ^= .44), and there was a significant interaction between the two factors (*F*[2.56, 28.33] = 3.57, *p* = .032, η*_p_*^2 ^= .25), likely owing to the near absence of segregation reported for 0° ΔIPD, regardless of envelope shape.

Pairwise comparisons indicated a significant difference in segregation across all four ΔIPDs (*p* < .005 in all cases, except between 180° and 90° (*p* = .018, difference = 10.0%)). *A posteriori* tests also indicated that significantly less segregation was reported for the FA-S-FR envelope than either the FA-SR (*p* = .006, difference = 20.2%) or SA-FR (*p* = .002, difference = 8.4%) envelope shapes. The difference between the FA-SR and SA-FR envelope shapes just failed to reach significance (*p* = .052, difference = 11.8%). All applicable statistics remained consistent at the *p* < .05 level when the 0° ΔIPD conditions were excluded from analysis—except the ΔIPD × envelope interaction, which became non-significant (*F*[2.23, 24.55] = 0.956, *p* = .407, η*_p_*^2 ^= .08) and the pairwise comparison between the FA-SR and SA-FR envelope conditions, which became significant (*p* = .043, difference = 16.1%).

### Discussion

The influence of ΔF in task 1A was consistent with previous reports (Bregman & Campbell, 1971; Miller & Heise, 1950; van Noorden, 1975), as was the lack of influence of envelope shape on ΔF-based segregation judgments (Roberts & Haywood, 2023; Singh & Bregman, 1997). Stream segregation increased with ΔIPD in task 1B. Although we did not assess lateralization judgments directly, it seems plausible that the strength of ΔIPD-based segregation may be influenced by the difference in perceived lateralization between A and B tones. Although perceived lateralization correlates better with ITD than IPD (Zhang & Hartmann, 2006), it is nevertheless the case that, for the 400-Hz stimuli tested here, lateralization should overall increase as IPD increases from 0° up to ±90° (Yost, 1981, who measured the lateralization of a 500-Hz tone). The notion that more segregation should be heard as the difference in perceived lateralization increases between the A and B subsets is consistent with the proposal that the perceived dissimilarity between subsets of sounds is the key factor determining the likelihood of stream segregation (Moore & Gockel, 2002, 2012). Sach and Bailey (2004) reached a similar conclusion after measuring ΔITD- and ΔILD-based streaming in an objective rhythmic masking-release task. In that study, ITD and ILD cues were varied independently, and performance was best predicted from the difference in perceived spatial position between A and B tones, as opposed to the absolute physical difference in either cue. Note, however, that in the current case of the 180° ΔIPD, the A tones were presented at +90° and the B tones at −90°. Yost (1981) observed that tones with these IPDs are occasionally lateralized to the opposite side of the head from the ear presented with the leading phase. We saw no evidence of reduced segregation due to ambiguous lateralization percepts (including in experiment 2, which also tested stimuli with ±60° IPDs, corresponding to a 120° ΔIPD). As IPDs between ±90°–180° become increasingly ambiguous (Yost, 1981; Zhang & Hartmann, 2006), we assume that less stream segregation would occur for stimuli presented in this range, owing to a reduced perceptual difference between the A and B subsets (e.g., A = +120°, B = −120°).

In contrast to ΔF, envelope shape had a strong influence on ΔIPD-based segregation, with the greatest segregation reported in the FA-SR conditions. The limited extent of segregation in the FA-S-FR conditions is, of itself, consistent with the findings of Boehnke and Phillips (2005) and of Füllgrabe and Moore (2012), who used a similar envelope and observed only weak ΔITD streaming despite testing large ΔITD values (Boehnke & Phillips, 2005, ΔITD = ±600µs; Füllgrabe & Moore, 2012, ΔITD = up to ±2 ms). However, the robust segregation observed in the current FA-SR conditions does not support the general conclusion of those authors that ΔIPD is a weak cue for segregation. Instead, the current data suggest that FA-S-FR envelopes may be suboptimal for observing ΔITD/ΔIPD segregation. In contrast, Schadwinkel and Gutschalk (2010, 2011) and Carl and Gutschalk (2013) all reported strong ΔITD-based segregation for FA-S-FR envelope stimuli. While the use of tone complexes in these latter studies might account for this discrepancy, our experiment also differed from these studies by testing three envelope shapes. It is possible that, in the current ΔIPD task, listeners compared the strength of segregation across trials, and that the stronger segregation heard in the FA-SR trials reduced the tendency to report segregation in the SA-FR and FA-S-FR trials.

What might account for the effect of envelope shape on ΔIPD-based stream segregation? As previously outlined, ITD/IPD cues present near onset and during rapidly rising amplitude have been shown to make the greatest contribution to perceived lateralization (Dietz et al., 2013; Hartung & Trahiotis, 2001; Haywood et al., 2021; Stecker, 2018; Stecker & Bibee, 2014; Tollin & Henning, 1998, 1999; Wallach et al., 1949). However, the current FA-S-FR and FA-SR envelopes shared identical onset ramps but nonetheless differed greatly in perceived stream segregation, so the extent of stream segregation cannot be accounted for solely by stimulus onset characteristics. The envelope shapes used here also differed in their offsets, with only the FA-SR envelope having a more gradual, “slow” decrease in energy.

A range of studies have shown evidence for a contribution to perceived lateralization from ITD/IPD cues around abrupt offsets (Akeroyd & Bernstein, 2001; Diedesch & Stecker, 2015; Haywood, Undurraga, & McAlpine, 2021; Stecker & Bibee, 2014; Stecker & Brown, 2010; Stecker & Hafter, 2009; Tobias & Schubert, 1959; Zurek, 1980). In contrast, there is little evidence of such perceptual “weighting” of offsets when energy decreases more gradually, such as with relatively slow sinusoidal amplitude modulation (Dietz et al., 2013; Haywood et al., 2021; Haywood & McAlpine, 2020; Hu et al., 2017; Stecker, 2018). Therefore, it seems likely that IPD cues during/near the abrupt offset of the FA-S-FR and SA-FR envelopes contributed towards perceived lateralization, but those cues present during/near the gradual offset of the FA-SR envelope did not. If so, one might speculate that ΔIPD-based segregation may be contingent on the time between stimulus elements that receive perceptual “weighting” for localization. More specifically, the time between “weighted” elements may have been *greater* for the FA-SR (only onset weighted) than for the SA-FR and FA-S-FR envelopes (onset *and* offset weighted). Given that only the onsets were weighted during the FA-SR envelope, the greater temporal separation between weighted elements may have reduced any temporal integration of binaural cues between successive tones. The notion that binaural temporal integration may reduce stream segregation is considered further in experiment 2.

## Experiment 2

Having demonstrated that stimulus envelopes with a fast attack and slow release (FA-SR) provide for the strongest ΔIPD-based stream segregation, we employed this envelope shape in experiment 2 to explore the effects of varying the ISI on stream segregation based on ΔF and ΔIPD cues. Stream segregation based on spectral differences is known to be reduced at longer ISIs, which results in slower rates of presentation (van Noorden, 1975). We also explored the effects of varying ΔF and ΔIPD on stream segregation for a fixed ISI.

### Method

#### Listeners

Twelve normal-hearing listeners initially completed the experiment. Responses from two listeners in task 2A did not conform to the widely established effects of ΔF and ISI on segregation judgments (e.g., van Noorden, 1975), and so the data from these listeners were excluded from analysis, and another two listeners were recruited to replace them. Of the 12 final listeners, six had previously completed experiment 1, one was tested at the University of Cambridge, five were male, and the mean age was 23.1 years (range = 18–31 years).

#### Stimuli

Tone duration was fixed at 80 ms and all tones were shaped with an FA-SR envelope (see experiment 1). Across conditions, the ISI was set to 0, 20, 40, or 60 ms. The duration of the silent pause (“–”) following each ABA triplet was scaled to preserve an equal onset-to-onset time between all A tones within the sequence (silent pauses of 80, 120, 160, or 200 ms, respectively, with increasing ISI). Experiment 2 was divided into three tasks.

#### Task 2A: ΔF

As for task 1A, the A tones were fixed at 400 Hz, and the B tones were set to 4, 6, or 8 ST higher. The signal presented to each ear was identical (diotic). The combination of four ISIs and three ΔFs led to 12 unique conditions.

#### Task 2B: ΔIPD

As for task 1B, both A and B tones were set to 400 Hz. In a control case, both A and B tones had an IPD of 0° (no ΔIPD). For the remaining three levels of ΔIPD, the A tones were presented with a right-ear-leading IPD, and the B tones with a left-ear-leading IPD of the same size. These values were ±30°, ±60°, or ±90° (ΔIPDs of 60°, 120°, and 180°, respectively). IPDs of ±30°, ±60°, and ±90° corresponded to fine-structure ITDs of ±208, ±416, and ±625 µs respectively. The combination of four ISIs and four ΔIPDs led to 16 unique conditions.

#### Task 2C: ΔF and ΔIPD

In task 2C, the ISI was fixed at 60 ms—i.e., the longest value used in tasks 2A and 2B. The A tones were 400 Hz, and the B tones were set to 0, 4, 6, or 8 ST higher. The A and B tones were presented with IPDs of ±0°, ±30°, or ±90° (ΔIPDs of 0°, 60°, or 180°, respectively). Note that the use of fixed IPD values resulted in differences in corresponding fine-structure ITD across different B-tone carrier frequencies. Specifically, ITD magnitude falls with increasing carrier frequency for a fixed IPD (at 400 Hz/0 ST, IPDs of +30° and +90° resulted in ITDs of 208 and 625 µs; 504 Hz/+4 ST = 165 and 496 µs; 566 Hz/+6 ST = 147 and 442 µs; 635 Hz/+8 ST = 131 and 394 µs). The combination of four ΔFs and three ΔIPD led to 12 unique conditions.

#### Procedure

The procedure was identical to that used in experiment 1. Training on each task again comprised two blocks, and each main task again comprised 20 blocks—corresponding to 240 trials in tasks 2A and 2C, and 320 trials in task 2B. The three tasks were presented in fully counterbalanced order across listeners, two rotations of the six possible permutations. Listeners completed the experiment in a single session, which typically took around 75 min.

### Results

#### The Effect of ISI on Stream Segregation (Tasks 2A and 2B)

**Figure 2.**
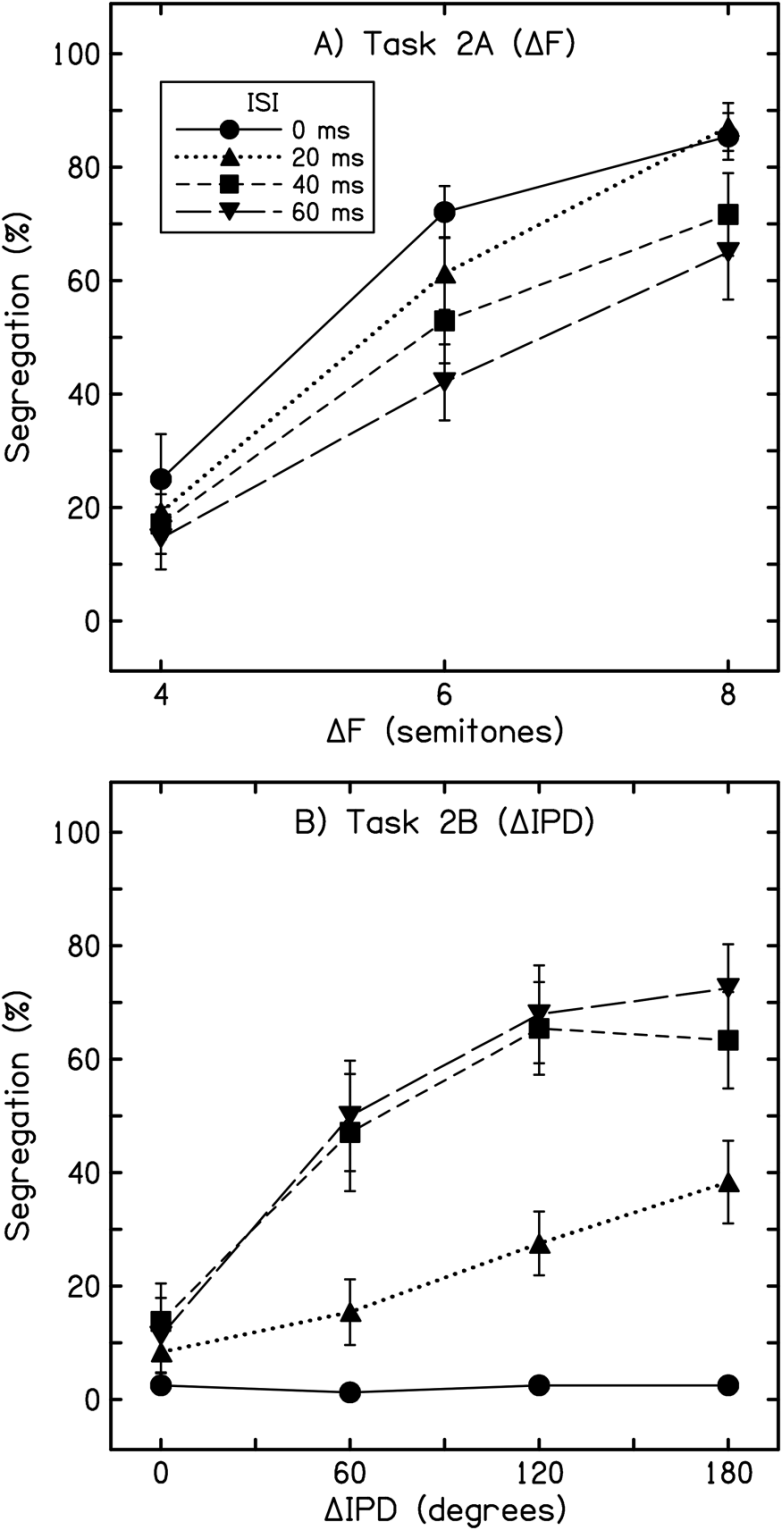
Results from experiment 2 (tasks 2A and 2B). Values are the overall percentage of segregated responses from 12 listeners. Error bars display ±1 intersubject standard error. (A) Results from task 2A: ΔF is plotted along the abscissa, and each trace represents a different ISI (see inset). (B) Results from task 2B: ΔIPD is plotted along the abscissa. Note the reversal of the effect of increasing ISI across the two tasks.

The exclusion and replacement of two listeners based on their performance in task 2A (ΔF) was noted above. Another two listeners reported very little ΔIPD-based segregation in task 2B, indicating segregation in only 0.3% and 12.5% of all trials, respectively, which was far below that reported by all other listeners. These listeners were not excluded, however, as both showed typical responses in the ΔF task. Data from the final 12 listeners were aggregated and averaged in the manner described for experiment 1 and are plotted in Figures 2 (tasks 2A and 2B) and 3 (task 2C).

**Figure 3.**
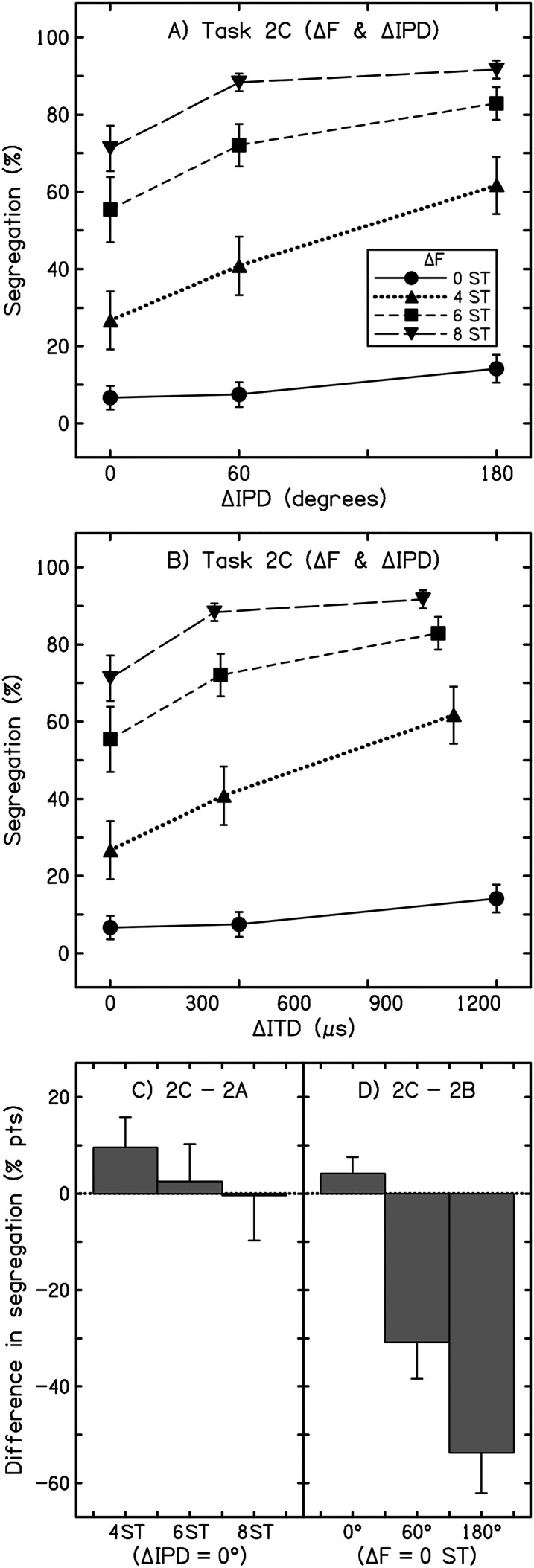
(A) Results from experiment 2 (task 2C). Values are the overall percentage of segregated responses from 12 listeners. Error bars display ±1 intersubject standard error. ΔIPD is plotted along the abscissa, and each trace represents a different ΔF. (B) As for (A), except results are plotted by ΔITD. (C) The change in reported segregation between identical ΔF conditions from task 2A to 2C (2C–2A). Each bar shows the change in mean segregation for a single condition, as labeled along the abscissa, and the error bars indicate the standard error of the difference in reported segregation in percentage points (% pts). (D) as for (C), except that the change between identical ΔIPD conditions from task 2B to 2C is shown (2C–2B).

As expected, ΔF-based segregation increased significantly with increasing ΔF (*F*[1.32, 14.50] = 34.66, *p* < .001, η*_p_*^2 ^= .76) and shorter ISIs (*F*[1.28, 14.08] = 6.70, *p* = .016, η*_p_*^2 ^= .38), and the interaction between these two factors was also significant (*F*[3.08, 33.86] = 3.95, *p* = .016, η*_p_*^2 ^= .26) (task 2A, Figure 2A). Pairwise comparisons indicated a significant difference in segregation between all three ΔFs when averaged across ISI (all permutations of comparison, *p* < .001). When averaged across ΔF, most comparisons between ISIs reached significance (*p* < .05). Only two did not, the comparison between 0 and 20 ms (*p* = .248, difference = 5.0%) and that between 0 and 40 ms, which approached but did not reach significance (*p* = .063, difference = 13.6%).

For stream segregation based on ΔIPD (task 2B, Figure 2B), listeners reported significantly more segregation for larger ΔIPDs (*F*[1.48, 16.25] = 30.38, *p* < .001, η*_p_*^2 ^= .73), similar to the effect of ΔF. In contrast to ΔF, however, stream segregation based on ΔIPD was less commonly reported at shorter ISIs (*F*[2.02, 22.16] = 20.50, *p* < .001, η*_p_*^2 ^= .65). The interaction between ΔIPD and ISI was also significant (*F*[3.87, 42.58] = 14.71, *p* < .001, η*_p_*^2 ^= .57), likely because little to no segregation was reported at 0° ΔIPD irrespective of ISI (as would be expected, because all tones were identical), as well as the low extent of segregation reported across all ΔIPDs at the shortest ISI (0 ms). Pairwise comparisons revealed a significant difference in segregation between all ΔIPDs (all permutations of comparison, *p* < .005), except between ΔIPDs of 120° and 180° (*p* = .144, difference = 3.0%). Significantly less segregation was reported for the 0-ms ISI compared to all three longer ISIs (three comparisons, *p* < .005 in all cases). The difference between ISIs of 20 and 40 ms and 20 and 60 ms also reached significance (*p* = .006, difference = 12.4%, and *p* = .021, difference = 15.7%, respectively), but there was no significant difference in reported stream segregation between the 40- and 60-ms ISI conditions (*p* = .289, difference = 3.3%).

#### Stream Segregation in a Task Containing Both ΔIPD and ΔF Cues (Task 2C)

We next explored how ΔF and ΔIPD were weighted for stream segregation when the two cues were presented together or in isolation within the same task (task 3C, Figure 3A). Significantly more segregation was heard both at larger ΔFs (*F*[2.11, 23.18] = 88.75, *p* < .001, η*_p_*^2 ^= .89) and at larger ΔIPDs (*F*[1.27, 13.96] = 23.78, *p* < .001, η*_p_*^2 ^= .68). However, even very large differences in IPD were insufficient to generate segregation when A and B subsets were identical in frequency (i.e., ΔF = 0 ST). For a ΔF of 8 ST, segregation did increase with ΔIPD, albeit only modestly (though this may in part reflect a ceiling effect as responses reached 91.66% segregated). Notably, however, ΔIPD had a relatively large effect on segregation for a ΔF of 4 ST, where reported segregation increased from around 26.66% of trials for a ΔIPD of zero to 61.66% for a ΔIPD of 180°. The interaction between the two cues for segregation was significant (*F*[3.62, 39.83] = 4.67, *p* = .004, η*_p_*^2 ^= .30), and pairwise comparisons found a significant difference between all four ΔFs (all permutations of comparison, *p* < .001), and between all three ΔIPDs (all permutations of comparison, *p* < .005).

As acknowledged above, the use of a fixed IPD resulted in differences in fine-structure ITD across carrier frequencies. To illustrate the consequences of this approach and its implications for lateralization, the results are replotted as a function of ΔITD in Figure 3B. Here, it is worth noting that the A tones were presented at 400 Hz and were right-leading in all conditions—only the B tones, which were always left-leading, were varied in frequency. As perceived lateralization is correlated with ITD (Zhang & Hartmann, 2006), this presumably led to instances where the B tones, when presented at higher frequencies, were less lateralized than their A-tone counterparts. Although this means that some caution is needed when interpreting the results—for example, potentially the seemingly greater influence of ΔIPD at 4-ST ΔF—our main conclusion from this experiment, concerning the overall lack of segregation in the 0-ST ΔF conditions, is unaffected by this aspect of the method.

In the mixed-cue task used here, a subset of conditions was identical to that presented in tasks where either cue was presented alone—i.e., task 2A had conditions with no ΔIPD, and ΔFs of 4, 6, and 8 ST, and task 2B had conditions with no ΔF and ΔIPDs of 0°, 60°, and 180°. Strikingly, in the single-cue task (2B), the 180° ΔIPD condition was reported predominantly as segregated (71.4%; ISI = 60 ms), but this same stimulus was reported predominantly as integrated (14.1%) in the task containing both ΔF and ΔIPD cues (task 2C). The size of this difference is highlighted by the contrast with the ΔF-only conditions, where segregation for the identical stimulus configuration was only marginally greater in the mixed-cue task than in the single-cue task. Comparisons of reported segregation across tasks 2A to 2C are illustrated in Figures 3C and D. We consider the substantial reduction in reported ΔIPD-based segregation from task 2B to 2C primarily to reflect the presence of ΔF cues in the latter task (i.e., a contextual effect, see discussion). Although across-frequency changes in ITD may have exerted a minor-to-moderate influence on reported segregation, it would seem unlikely that this factor alone could account for the large across-task differences observed (i.e., Figure 3D).

### Discussion

The overall influences of ΔF and ISI on segregation in the ΔF task (2A) were consistent with previous observations (Bregman & Campbell, 1971; Miller & Heise, 1950; van Noorden, 1975). However, to our knowledge, only one previous study has examined the influence of ISI on ΔITD- or ΔIPD-based segregation. Boehnke and Phillips (2005) measured subjective streaming for bursts of noise containing ITDs of ±600 µs (ΔITD = 1200 µs). The duration of each noise burst was fixed at 90 ms, and the ISI was set to either 30 or 90 ms. However, little segregation was reported in either case—a finding that may be due to the form of the envelope these authors employed (effectively the FA-S-FR envelope used in experiment 1) and/or contextual effects (as discussed below). In the current experiment, ΔIPD-based segregation was greater at relatively slow presentation rates (longer ISIs)—the opposite of the pattern seen for ΔF-based segregation (task 2A; Bregman & Campbell, 1971; Miller & Heise, 1950; van Noorden, 1975). Further, while ISI had a significant influence on segregation based on both ΔF and ΔIPD cues, it exerted a greater influence in the ΔIPD task. For example, the two largest ΔIPD conditions (120°, 180°) were nearly always heard as integrated when the ISI was 0 ms (<15% segregation, Figure 2B), but were heard predominantly as segregated at longer ISIs (>60% segregated at ISIs of 40–60 ms, Figure 2B).

The influence of ISI in the ΔIPD task may reflect the temporal integration of binaural information across the A and B subsets. We propose that: (1) across-subset binaural temporal integration should increase at shorter ISIs, and (2) that this may reduce the extent of perceived stream segregation. Concerning the first proposal, ITD cues have been shown to be integrated across time, and in a manner often conceptualized as a processing window of fixed duration, extending backward from the current moment. The output of this window corresponds to an average of the binaural information within. Estimates of window duration—variable and changing with task demand, experimental procedure, and individual performance—commonly range from 50 to 250 ms (Akeroyd & Summerfield, 1999; Culling & Summerfield, 1998; Grantham & Wightman, 1978, 1979; Holube et al., 1998; Kollmeier & Gilkey, 1990). However, the binaural system is sensitive to very brief changes in ITD, or rapid oscillations in interaural parameters in certain listening tasks (e.g., Bernstein et al., 2001; Siveke et al., 2008). This has led to the proposal that the binaural system is subject to “sluggish” long-duration temporal integration in tasks that require the higher-level re-estimation of binaural parameters, as opposed to the rapid sensory encoding of ITD/IPD necessary for detection (Eurich & Dietz, 2023; Yost, 1985).

Concerning the proposal that binaural temporal integration may reduce the extent of perceived stream segregation, binaural “sluggishness” has been associated with impaired performance in binaural unmasking (Culling & Summerfield, 1998; Kollmeier & Gilkey, 1990), tracking moving sound sources (Perrott & Musicant, 1977), understanding speech in noise (Culling & Colburn, 2000), and localizing consecutive sounds (Perrott & Pacheco, 1989; Strybel & Fujimoto, 2000). Concerning stream segregation, Simon and Winkler (2018) measured ΔF-based segregation for 10-ms pure tones with ISIs between 0 and 350 ms. Note that this tone duration is much shorter than is usual for stream segregation stimuli, which also resulted in very rapid presentation rates at short ISIs. As would be expected from previous research, the authors found reduced segregation at long compared to short ISIs (>100 ms, see also Bregman & Campbell, 1971; Miller & Heise, 1950; van Noorden, 1975). More surprisingly, segregation also decreased at short ISIs (<50 ms), such that segregation was most apparent for ISIs between 50 and 100 ms. Simon and Winkler (2018) proposed that, for very rapid presentation rates, (monaural) temporal integration between A and B subsets promoted the grouping of adjacent short sounds, leading to a stronger perception of integration. Presumably, temporal integration would act to “smear” information across the A and B subsets of the streaming stimulus, and so reduce the basis for their segregation. In our ΔF task, and unlike Simon and Winkler (2018), we observed increased segregation at the shorter ISIs tested (range = 0–60 ms). This difference likely reflects the use of much longer tones than those used by Simon and Winkler (2018)—80 ms versus 10 ms—and consequently the slower overall presentation rate in the current experiment. However, as binaural temporal integration is generally accepted to operate over longer timescales than monaural temporal integration, it is plausible that a mechanism similar to that proposed by Simon and Winkler (2018) may have been evident in the current ΔIPD task—namely that binaural temporal integration between successive A and B subsets promoted perceptual integration.

It is reasonable to question whether it is possible to estimate the time constant for binaural temporal integration acting in the present stream segregation task. Reported segregation did not change greatly between ISIs of 40 and 60 ms, suggesting that binaural integration did not occur between successive tones at these separations. However, some caution is required, as the tendency for reduced segregation at longer ISIs widely observed in ΔF stimuli may be a general grouping principle that also affects ΔIPD stimuli. In other words, at a sufficiently long ISI, across-subset binaural temporal integration may be reduced or absent, but the listener may nonetheless perceive integration because of a general mechanism that reduces the tendency for segregation at slower presentation rates. We speculate that such a mechanism would become apparent at ISIs >60 ms; it would seem likely that at some duration of ISI, the tendency to hear ΔIPD stimuli as segregated would inevitably fall.

The studies by Schadwinkel and Gutschalk (2010, 2011), and by Carl and Gutschalk (2013) all presented repeating “ABBB….” sequences of complex-tone stimuli, for which the tone duration was 125 ms and there was no ISI. Despite this, all these studies found strong ΔITD-based segregation. One possibility, therefore, is that temporal factors besides ISI—such as sound duration and/or overall presentation rate (onset-to-onset time)—may have influenced the extent of ΔITD-based segregation in these studies. In the current ΔIPD-based stimulus arrangement, the 40-ms ISI conditions resulted in a 120-ms onset-to-onset time, which was similar to the referenced studies and also resulted in a strong degree of segregation. For ΔF-based segregation, the ISI between sounds within the same frequency regions (i.e., within either the A or B subset) is considered the primary temporal factor that influences segregation (Bregman et al., 2000), but it is unknown whether this is also the case for ΔITD and/or ΔIPD-based segregation. Further research would be needed to distinguish between these temporal factors and the extent to which binaural temporal integration can account for ΔITD and/or ΔIPD-based segregation in sequences with different temporal arrangements. A second consideration is that Schadwinkel and Gutschalk (2010, 2011) and Carl and Gutschalk (2013) presented complex-tone stimuli with a consistent ITD across frequencies. Such stimuli would likely provide more robust cues for lateralization than the current pure tones varying only in IPD, as (a) they contained a time-of-onset (and offset) ITD and (b) there is evidence for a summation of information across components of a complex tone that improves ITD detection (Buell & Hafter, 1991). Further study would be required to quantify differences in stream segregation arising from ΔIPD and ΔITD cues (the latter including complex tones).

#### Contextual Effects

ΔF and ΔIPD cues were each presented in isolation in tasks 2A and 2B, respectively (“single cue” tasks), but both cues were present in task 2C (“mixed cue”). In task 2C, segregation increased when both cues were presented together—that is, for ΔFs of 4 ST or greater, reported segregation increased when a ΔIPD was also present. This is consistent with the notion that stream segregation is determined by the perceptual dissimilarity between A and B tones (Moore & Gockel, 2002, 2012). Two tones that differ in both frequency and IPD should have an overall greater perceptual dissimilarity than two tones that differ only in one dimension. Indeed, for the 4-ST conditions of task 2C, a ΔF that was not influenced by ceiling effects (Figure 3), increasing ΔIPD led to an appreciable rise in segregation—more so than in the conditions where no ΔF was present (0 ST). In the context of this mixed-cue task, ΔIPD was most effective at promoting segregation when a frequency cue was also present. In other words, although ΔIPD alone did not strongly promote segregation in this task, the cue did have an additive effect towards segregation when accompanying a frequency-difference cue.

When no ΔIPD was present in the mixed-cue task (2C), stream segregation increased with ΔF in a manner consistent with the single-cue task (2A). However, when only a ΔIPD was present in the mixed-cue task, sequences were heard primarily as integrated, irrespective of the magnitude of the ΔIPD. This result was inconsistent with clear ΔIPD-based segregation in the corresponding single-cue task (2B), despite both tasks testing the same listeners with an identical subset of conditions. This outcome suggests that the difference in results reflects a contextual effect. Previous research on ΔF-based streaming has shown that reported segregation on a given trial is decreased when ΔF in preceding trials (1-, 2-, or 3-back) was large, indicating that any given current segregation judgment is made within the context of prior stimuli recently heard (Snyder et al., 2008, 2009; Snyder & Weintraub, 2013). Potentially, in the mixed-cue task (2C), segregation judgments were influenced strongly by prior ΔF context, such that little-to-no segregation was reported in the 0-ST conditions for the task where 75% of conditions included some frequency difference (4, 6, or 8 ST). By comparison, ΔIPD context seemingly exerted far more influence on segregation judgments in the task where ΔF cues were absent (2B). This may suggest a form of “hierarchical” weighting of cues in present and prior stimuli—in task 2C, ΔF may have been a more dominant cue than ΔIPD, such that listeners made judgments primarily based on ΔF. Such a hierarchy of contextual effects could also account for discrepancies between the outcomes of previous studies. Both Boehnke and Phillips (2005) and Füllgrabe and Moore (2012) presented forms of mixed-cue subjective tasks and observed only weak ΔITD-based segregation. In contrast, Schadwinkel and Gutschalk (2010, 2011), and Carl and Gutschalk (2013) tested single-cue (ΔITD) tasks and observed more marked ΔITD-based segregation.

## Experiment 3

We next assessed stream segregation using an objective temporal discrimination task in which stimuli comprised 8 × ABA– triplets. A progressive delay was imposed on the final four B tones while the timing of the A tones was held constant (see Roberts et al., 2002; Vliegen et al., 1999). Delay detection is less challenging when integration is heard, as the unified rhythm perceived becomes anisochronous, but more challenging when segregation is heard, as the timing of the B tones relative to their neighboring A tones becomes much less salient. Therefore, poor performance is an indication of obligatory stream segregation—i.e., segregation that occurred despite being detrimental to performance. ΔITD appears to have little impact on temporal discrimination thresholds, suggesting that ΔITD *per se* does not promote obligatory segregation (Boehnke & Phillips, 2005; Füllgrabe & Moore, 2012, 2014; Stainsby et al., 2011). However, as our preceding experiments found that an FA-SR envelope is more effective than an FA-S-FR envelope at promoting segregation (experiment 1), and that segregation increases with ISI (at least within the range tested in experiment 2), experiment 3 re-evaluated temporal discrimination performance in sequences optimized for ΔIPD-based segregation. Note that experiment 3 presented conditions with either a ΔF, a ΔIPD, or a combination of both segregation cues. Given that the task required the objective detection of temporal differences, and conditions were tested individually in a staircase procedure, it was assumed that contextual effects would have less bearing on performance than was observed in experiment 2.

### Method

#### Listeners

Twelve normal-hearing listeners completed the experiment. Three had previously completed experiment 1, two had completed experiment 2, and two had completed both. Six listeners were tested at the University of Cambridge, four were male, and the mean age was 24.0 years (range = 19–29 years).

#### Stimuli

All tones were 80 ms long and shaped with an FA-SR envelope (70 dB SPL). Excluding any delay, the default ISI was 60 ms, and the longer silence (“–”) was always 200 ms. Each sequence comprised eight ABA– triplets (4.48 s total duration). In a reference sequence, the ISI remained at 60 ms in all eight triplets. In a target sequence, a delay was imposed on the final four triplets. Here, each B tone was delayed by extending the preceding ISI (A-to-B in ABA–) and reducing the following ISI (B-to-A in ABA–). This meant that the onset-to-onset time between successive A tones was held constant throughout the test sequence, and identical to that for the reference sequence. The delay was imposed progressively over the 5th–8th triplets (5th triplet = 25% of max. delay, 6th = 50%, 7th = 75%, and 8th = 100%). The size of the delay was varied in an adaptive procedure, and subsequent threshold measurements are given for the maximum delay present in the sequence—i.e., that present in the 8th ABA– triplet.

**Figure 4.**
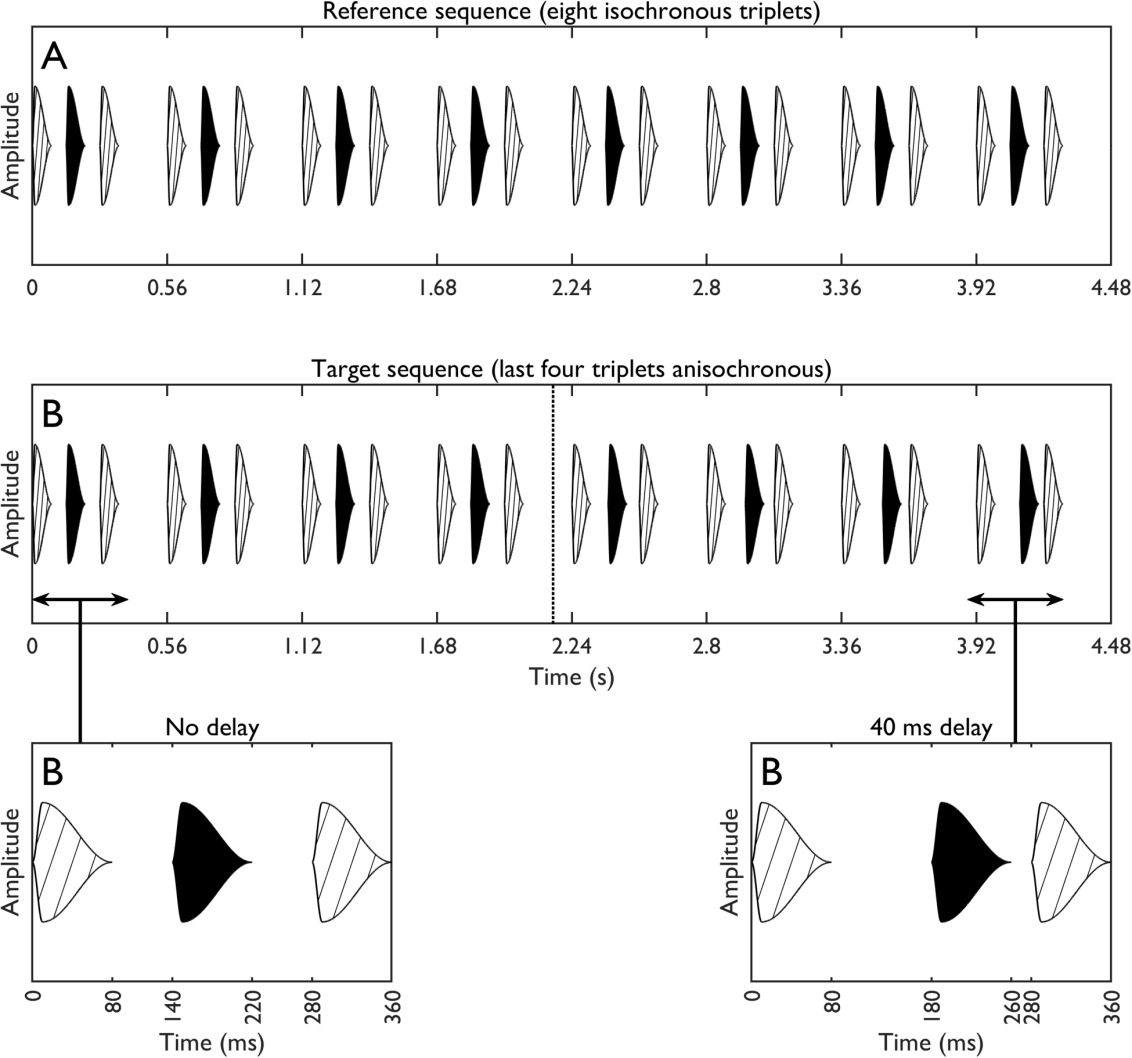
Stimuli from experiment 3. (A) A reference sequence comprising eight isochronous triplets, in which the B tones are evenly spaced between neighboring A tones. (B) A target sequence, in which only the first four triplets are isochronous. A delay is imposed progressively on the B tones in the final four triplets (i.e., the maximum delay occurs in the final triplet). The two lower panels provide an expanded view of the first and last triplets.

Figure 4 provides a schematic of the target and reference sequences—here, the target sequence contains the largest delay tested (40 ms). Note that at the maximum delay of 40 ms, there remained a 20-ms ISI between the delayed B tone and the following A tone. This contrasts with most previous temporal discrimination procedures, where the maximum delay resulted in a 0 ms B-to-A ISI (Boehnke & Phillips, 2005; Füllgrabe & Moore, 2012, 2014, Roberts et al., 2002; Stainsby et al., 2011; Vliegen et al., 1999). This modification was made because experiment 2 found that listeners reported ΔIPD-based segregation was greatly reduced for a 0-ms ISI in comparison to a 20-ms ISI (Figure 2B). The A tones were set to 400 Hz, and ΔF was varied across conditions (0, 4, or 6 ST). In addition, the ΔIPD was set to either 0° (diotic) or 180° (A = + 90°, B = −90°; A = + 625µs fine-structure ITD, B = −625, −496, or −442 µs at ΔFs of 0, 4, or 6 ST, respectively), the latter typically being the ΔIPD which resulted in the greatest proportion of segregation judgments in experiments 1 and 2. The combination of three ΔFs and two ΔIPDs led to a total of six unique conditions.

#### Procedure

An adaptive two-interval, two-alternative forced-choice procedure was used. A trial comprised one interval containing a target sequence and the other containing a reference sequence. A 400-ms silence separated the two intervals. The presentation order was randomized, and listeners were asked to identify which interval contained the target sequence. Listeners responded via the computer keyboard, and on-screen visual feedback was provided to indicate a correct or incorrect response. There was a 400-ms silence between the keyed response and the automatic onset of the next trial.

A two-up, one-down adaptive staircase procedure was used to estimate the 70.7% correct point on the psychometric function (Levitt, 1971). Each adaptive run measured performance for a single stimulus condition. The staircase began with the maximum possible cumulative delay (40 ms), which was reduced for correct performance. From this maximum, the delay was adjusted by a factor of 1.414, reducing to 1.189 after two turn points. This procedure ran until listeners completed ten reversals, at which point the run ended. Runs terminated if listeners made six successive incorrect responses at the maximum delay (40 ms) before making two successive correct responses (after which this count was reset).

A test block comprised one run of each of the six conditions, presented in shuffled order. Listeners completed three blocks, across two or three test sessions. An additional run was completed to replace any run where the log standard deviation of the turn points exceeded 0.2 (occurring in 28 out of 216 possible instances). Similarly, an additional run was completed for any condition where the across-run threshold estimates exceeded a log standard deviation of 0.2, and the outlying threshold estimate was replaced (occurring in eight out of 72 possible instances). The experiment typically took two hours to complete.

### Results

**Figure 5.**
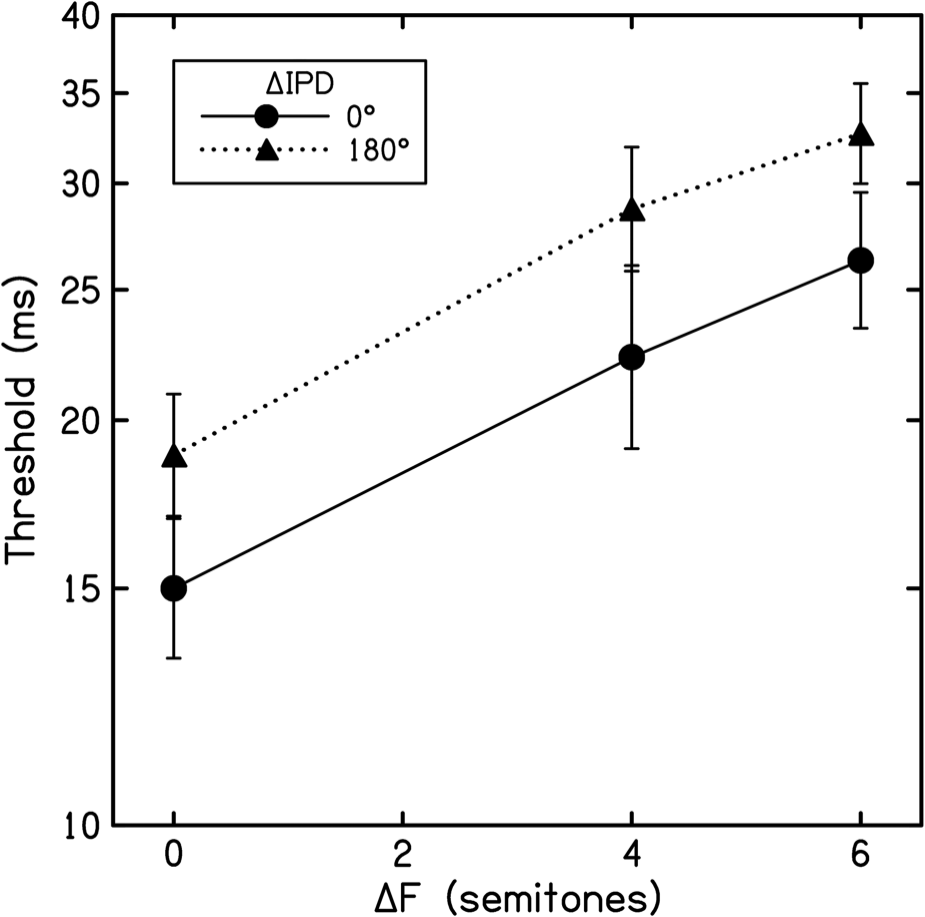
Results from experiment 3. Each point represents the average threshold for the detection of delayed B tones for 12 listeners. ΔF is plotted along the abscissa, and each trace represents a different ΔIPD (see inset). Error bars display ±1 intersubject standard error.

The experiment measured the detection of delayed tones in a task where the perception of stream segregation was expected to impair performance. For each listener, thresholds from each run of the same condition were averaged to yield a geometric mean threshold. Mean thresholds were then averaged across listeners to yield a grand mean for each condition (Figure 5). Thresholds increased with both ΔF and ΔIPD, an indication that both factors promoted stream segregation. A two-way repeated measures ANOVA confirmed significant main effects of both ΔF (*F*[1.34, 14.70] = 25.81, *p* < .001, η*_p_*^2 ^= .70) and ΔIPD (*F*[1, 11] = 24.71, *p* < .001, η*_p_*^2 ^= .69). The interaction between these two factors was not significant (*F*[1.71, 18.84] = 0.20, *p* = .786, η*_p_*^2 ^= .02). Overall, the 180° ΔIPD resulted in thresholds rising by a factor of 1.26.

### Discussion

Overall, the data from experiment 3 (Figure 5) confirm previous findings. Thresholds increased with ΔF (Roberts et al., 2002; Vliegen et al., 1999), and increased further with ΔIPD. The modest, but significant detrimental effect of ΔIPD on delay detection thresholds is broadly consistent with previous studies (Boehnke & Phillips, 2005; Füllgrabe & Moore, 2012; Stainsby et al., 2011). Boehnke and Phillips (2005) presented a broadband noise stimulus and, among other conditions, in one binaural configuration the A sounds were set to −600 µs ITD and the B sounds to +600 µs ITD. The presence of this ΔITD increased thresholds by a factor of 1.13 relative to the diotic control case, an elevation that was not significant (the authors directly reported average thresholds of 9.51 ms [diotic] and 10.76 ms [ΔITD] in their results). The observed effect of ΔIPD in experiment 3 was more robust—i.e., larger, and statistically significant—compared to the influence of ΔITD observed by Boehnke and Phillips (2005). Although some aspects of our results for the 180° ΔIPD may have been influenced by decreasing ΔITD with increasing ΔF, we observed that this ΔIPD promoted a similar extent of segregation at each ΔF tested—raising thresholds by a factor of 1.26, 1.29, and 1.24 at ΔFs of 0, 4 and 6 ST, respectively. This would appear consistent with Füllgrabe and Moore's (2012) observation that performance varies little once ΔITD extends beyond 500 µs, which was the case for all the current 180° ΔIPD stimuli.

Stainsby et al. (2011) presented band-pass filtered noise and used ΔITD in a similar way to Boehnke and Phillips (2005), but tested ITDs in the range ±500 µs to ±2 ms. In the experiment most similar to ours (Stainsby et al., 2011, experiment 2), the A and B subsets either shared an identical passband noise or differed in the passband (353–707 Hz vs. 500–1000 Hz). At the group level, those authors observed a monotonic increase in thresholds with ΔITD by a factor of 2.0 with no passband difference and a factor of 1.3 with a passband difference (both factors for diotic vs. 2-ms ΔITD). In contrast, there was no evidence of a reduction in the effect of ΔIPD with increasing ΔF in the current experiment. David et al. (2015) also measured temporal discrimination for broadband noise and reported that a 1-ms ΔITD (±500 µs) raised thresholds by a factor of around 2 (as estimated from their results [David et al., Figure 6]). Finally, Füllgrabe and Moore (2012) adopted the paradigm of Stainsby et al. (2011) but presented pure-tone stimuli (500 vs. 707 Hz), reporting that thresholds increased up to a ΔITD of 500 µs, but beyond that changed little, or even slightly decreased. These authors proposed that this may have reflected the lack of ongoing envelope ITD cues in their pure-tone stimuli, unlike the noise signal used by Stainsby et al. (2011). A second experiment assessed a narrower range of ΔITD conditions, up to a maximum of 500 µs. We estimate from their data figure that a ΔITD of 500 µs resulted in thresholds rising by a factor of about 1.2 in those conditions with no ΔF. ΔITD had seemingly less effect when a ΔF was present, likely reflecting at least in part a ceiling effect from the maximum detectable delay when listening to the B tones in isolation from the A tones (i.e., the level of performance achievable when complete segregation is heard). The authors later reported similar findings for their no-ΔF conditions when tested in a group of older normal-hearing listeners (Füllgrabe and Moore, 2014).

Overall, the current changes to stimulus parameters—the FA-SR envelope and extended ISIs—did not cause any noteworthy change in results in comparison to Füllgrabe and Moore (2012, 2014), who presented otherwise comparable pure-tone stimuli and found a modest but significant threshold elevation from ΔITD/ΔIPD. Except for Boehnke and Phillips (2005), other authors have reported larger effects of ΔITD using noise-burst stimuli (David et al., 2015; Stainsby et al., 2011). It is worth noting that most temporal discrimination studies of ΔITD (Boehnke & Phillips, 2005; David et al., 2015; Füllgrabe & Moore, 2012; Stainsby et al., 2011), and the current study concerning ΔIPD, assessed segregation in the context of a mixed-cue task (varying ΔITD/ΔIPD alongside either ΔF or other forms of binaural difference), but given the use of a staircase procedure, it is not expected that contextual effects would influence performance to any great extent. It merits comment, however, that almost all listeners reported difficulties in detecting the delay in the 180° ΔIPD stimuli on first hearing and great difficulty tracking the movement of the intracranial sound image. This difficulty was partly reduced over the first several presentations, such that listeners eventually demonstrated reasonably high levels of performance in the task. This suggests that listeners may have learned a strategy to minimize any stream segregation or distraction effects arising from the 180° ΔIPD, though it is difficult to quantify any such rapid learning effect from the current adaptive staircase procedure.

Both Boehnke and Phillips (2005) and Füllgrabe and Moore (2012) measured subjective stream segregation in combination with temporal discrimination performance and found that their listeners did not report strong subjective segregation from ΔITD cues. For this reason, these authors proposed that ΔITD is a relatively weak cue for subjective and obligatory (temporal discrimination) stream segregation. In contrast, we observed robust reported ΔIPD-based segregation in the subjective tasks of experiments 1 and 2. We, therefore, propose that ΔITD/ΔIPD can be effective for segregation in a suitably designed subjective task—one that accounts for critical features of the stimulus envelope, ISI, and the general context in which the cues are presented over the course of an experimental session.

## Concluding Remarks

In summary, this research has demonstrated that ΔIPD can promote subjective stream segregation and has identified three factors that influence this effect. First, in contexts where only IPD cues are present, reported segregation is greatest for FA-SR (damped) type envelopes. Second, ΔIPD-based segregation increases with increasing ISI, a trend opposite to ΔF-based segregation and which most probably reflects the sluggishness of binaural temporal integration. Third, ΔIPD-based segregation appears strongly influenced by contextual effects—i.e., by the characteristics of other stimuli present in the listening task. We have discussed how these factors may have influenced previous findings concerning subjective ΔITD-based segregation (Boehnke & Phillips, 2005; Carl & Gutschalk, 2013; Füllgrabe & Moore, 2012; Schadwinkel & Gutschalk, 2010, 2011). In particular, our data suggest that the failure to observe clear subjective ΔITD-based streaming in many previous studies may be a consequence of the specific stimulus parameters employed. We have demonstrated that IPD cues can only operate as effective cues for subjective stream segregation when sounds are presented with optimal envelope shapes, relatively long ISIs between consecutive sounds, and in an experimental context where ΔF cues are absent. Although we have generally assumed that our findings from ΔIPD stimuli are generalizable to ΔITD stimuli, there may be some benefit in testing this assumption explicitly in further work given that this cue (rather than ΔIPD per se) is considered the basis for perceived lateralization (Zhang and Hartmann, 2006), and because only ITDs occur in real-world listening.

We also measured temporal discrimination thresholds and observed that ΔIPD caused a modest but significant impairment in performance, which is consistent with previous literature that found ΔITD promotes only a moderate amount of obligatory stream segregation (Füllgrabe & Moore, 2012; Stainsby et al., 2011). These results suggest that listeners are able to elect (at least in part) to disregard spatial cues in circumstances where they are detrimental to desired auditory object formation—an ability that may be useful in real-world listening, such as when spatial cues are ambiguous or unreliable in a given listening environment.

The current experiments tested relatively short-duration streaming stimuli—sequences comprising five (experiments 1 and 2) or eight (experiment 3) ABA– triplets. The tendency to hear ΔF-based stream segregation increases, or “builds up,” over the course of several seconds after sequence onset (Anstis & Saida, 1985; Bregman, 1978; Haywood & Roberts, 2013), and Schadwinkel and Gutschalk (2010) reported build-up effects in their study of subjective ΔITD segregation. The current results likely reflect “early” segregation—that which was heard before the build-up is complete. As there has been no direct comparison between the dynamics of build-up for ΔF and ΔIPD (or ΔITD) stimuli, it may be the case that the rate or extent of build-up differs between these classes of stimuli. As such, aspects of the current results may not generalize to longer-duration stimuli, and further characterization of build-up effects in ΔIPD or ΔITD stimuli may be informative.

ΔITD cues have previously been classified as weak-to-moderate cues for stream segregation (e.g., see Moore & Gockel, 2012). Some aspects of our results are in accord with this interpretation—specifically, ΔIPD-based segregation can be greatly reduced by experimental context, and ΔIPD cues have only a moderate influence on temporal discrimination performance. However, the current results demonstrate that ΔIPD *can* be highly effective at promoting reported segregation in certain circumstances, and we highlight previous research that has shown ΔITD cues aid performance in objective tasks where segregation is beneficial to performance (Hartmann & Johnson, 1991; Middlebrooks & Onsan, 2012; Sach & Bailey, 2004). Therefore, we propose that ΔITD/ΔIPD cues are not necessarily weak cues for stream segregation but are instead *elective* cues, such that listeners are able to either use or largely ignore the cue depending on task demands, and such that subjective ΔIPD-based segregation appears highly prone to broader listening context.
